# Supplementary material for: Neutrophil serine proteases degrade endothelial cortactin and promote extravasation
Source: J Cell Biol. 2026 May 12;225(7):e202410019. doi: 10.1083/jcb.202410019 (PMC13163681; doi:10.1083/jcb.202410019)
Supplement: Table S1 — shows reagents and antibodies used in this study. [file jcb_202410019_tables1.docx]

**Supplementary Table 1.** Reagents and antibodies used in this study.

| Reagent | Source | Identifier |
| --- | --- | --- |
| Antibodies | | |
| Anti-cathepsin G | Invitrogen | Cat #PA5-99402 |
| Anti-CD31 (Clone 390) | eBioscience | Cat #16-0311-38 |
| Anti-cortactin (clone 289H10) | Home-made Lai *et al.*, Mol. Biol.  Cell, 2009 | |
| Anti-GAPDH (Clone 0411) | Santa Cruz | Cat #sc-47724 |
| Anti-MRP-14 (clone 2B10) | US Biological | Cat #376767 |
| Anti-γ Tubulin Monoclonal Antibody (Clone 4D11) | ThermoFisher Scientific | Cat #MA1-850 |
| APC/Cyanine7 anti-mouse Ly-6G Antibody (Clone 1A8) | Biolegend | Cat # 127624 |
| Goat Alexa Fluor 488 anti-mouse IgG (H+L) | Invitrogen | Cat #A-11001 |
| Goat Alexa Fluor 488 anti-rabbit IgG (H+L) | Invitrogen | Cat #A-11008 |
| Goat Alexa Fluor 568 anti-mouse IgG (H+L) | Invitrogen | Cat #A-11004 |
| Goat Alexa Fluor 647 anti-mouse IgG (H+L) | Invitrogen | Cat #A-21235 |
| Goat Alexa Fluor 647 anti-rabbit IgG (H+L) | Invitrogen | Cat #A-21244 |
| Goat anti-mouse IgG-HRP | Santa Cruz | Cat #sc-2005 |
| Goat anti-rabbit IgG-HRP | Santa Cruz | Cat #sc-2357 |
| Human TruStain FcX (Fc Receptor Blocking Solution) | Biolegend | Cat #422301 |
| Pacific Blue anti-human CD45 Antibody | Biolegend | Cat # 304022 |
| TruStain FcX™ PLUS (anti-mouse CD16/32) Antibody | Biolegend | Cat #156604 |
| Ultra-LEAF™ Purified anti-mouse Ly-6G/Ly-6C (Gr-1) Antibody (clone: RB6-8C5) | Biolegend | Cat # 108435 |
| Ultra-LEAF™ Purified Mouse IgG2b, κ Isotype Ctrl Antibody | Biolegend | Cat # 401216 |
| Cytokines, inhibitors, recombinant proteins and kits | | |
| AEBSF | Sigma | Cat #A8456-100MG |
| Alexa Fluor 488 Antibody Labeling kit | Invitrogen | Cat #A20181 |
| Alexa Fluor 555 Antibody Labeling kit | Invitrogen | Cat #A20187 |
| Alexa Fluor 647 Antibody Labeling kit | Invitrogen | Cat #A20186 |
| Alpha-1-antitrypsin (AAT) | Zemaira®, CSL Behring. Supplier: Solutesa S.A. de C.V. | |
| Calpain Inhibitor I, ALLN | Sigma | Cat #A6185-5MG |
| Human recombinant TNFα | PeproTech | Cat #300-01A |
| Leupeptin | Sigma | Cat #L2884-25MG |
| LTB4 | Merck | Cat # L0517-25UG |
| MG-132, Ready Made Solution | Sigma | Cat #M7449-1ML |
| Murine IL-1β | PeproTech | Cat # 211-11B |
| Murine KC (CXCL1) | PeproTech | Cat # 250-28 |
| Murine TNFα | PeproTech | Cat #315-01A |
| PD98059 | Sigma | Cat # P215-5MG |
| PMSF Roche | Sigma | Cat #10837091001 |
| Recombinant Human ICAM-1-Fc Chimera (carrier-free) | Biolegend | Cat # 552906 |
| Sivelestat sodium salt hydrate (ONO-5046) | Sigma | Cat #S7198-5MG |
| Other reagents | | |
| AF488 Phalloidin | Invitrogen | Cat #A12379 |
| BD Pharm Lyse™ | BD Bioscience | Cat #555899 |
| Endothelial Cell Medium (ECM) | ScienCell^TM^ Research Laboratories | Cat #1001 |
| Ethylenediaminetetraacetic acid (EDTA) | Sigma | Cat #E9884-500G |
| Fetal bovine serum | Biowest | Cat #S1810 |
| HEPES | HEPES | Cat #P5455-100GR |
| Histopaque-1077 (1.077 g/ml) | SIGMA | Cat #10771 |
| Histopaque-1119 (1.119 g/ml) | SIGMA | Cat #11191 |
| ProLong Gold mountant with Dapi. | Invitrogen | Cat #P36935 |
| Rhodamine Phalloidin | Invitrogen | Cat #R415 |
| RPMI-1640 Medium | Sigma | #R4130 |
| SuperSignal West Femto substrates | ThermoFisher, Scientific | Cat #34087 |
| Triton X-100 | Sigma | Cat #T9284 |
| TrypLE™ Express | Gibco | Cat #12604-013 |
| Trypsin-EDTA 0.25% | Sigma | Cat #T4049-500ML |
