# Supplementary material for: Neutrophil serine proteases degrade endothelial cortactin and promote extravasation
Source: J Cell Biol. 2026 May 12;225(7):e202410019. doi: 10.1083/jcb.202410019 (PMC13163681; doi:10.1083/jcb.202410019)
Supplement: SourceData F3 — is the source file for Fig. 3. [file jcb_202410019_sourcedataf3.pdf]

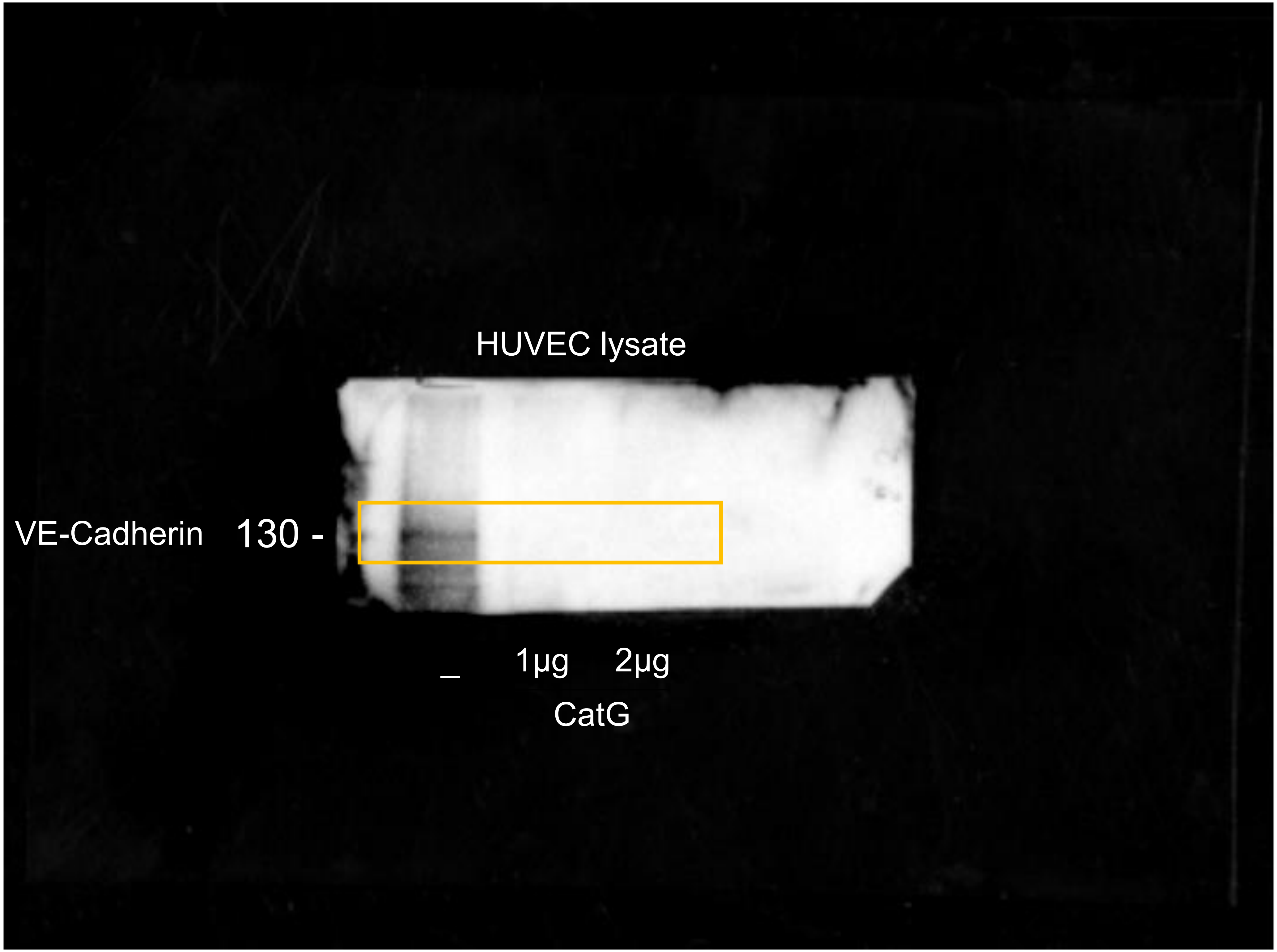
HUVEC lysate
VE-Cadherin
130 -
_
1µg
2µg
CatG

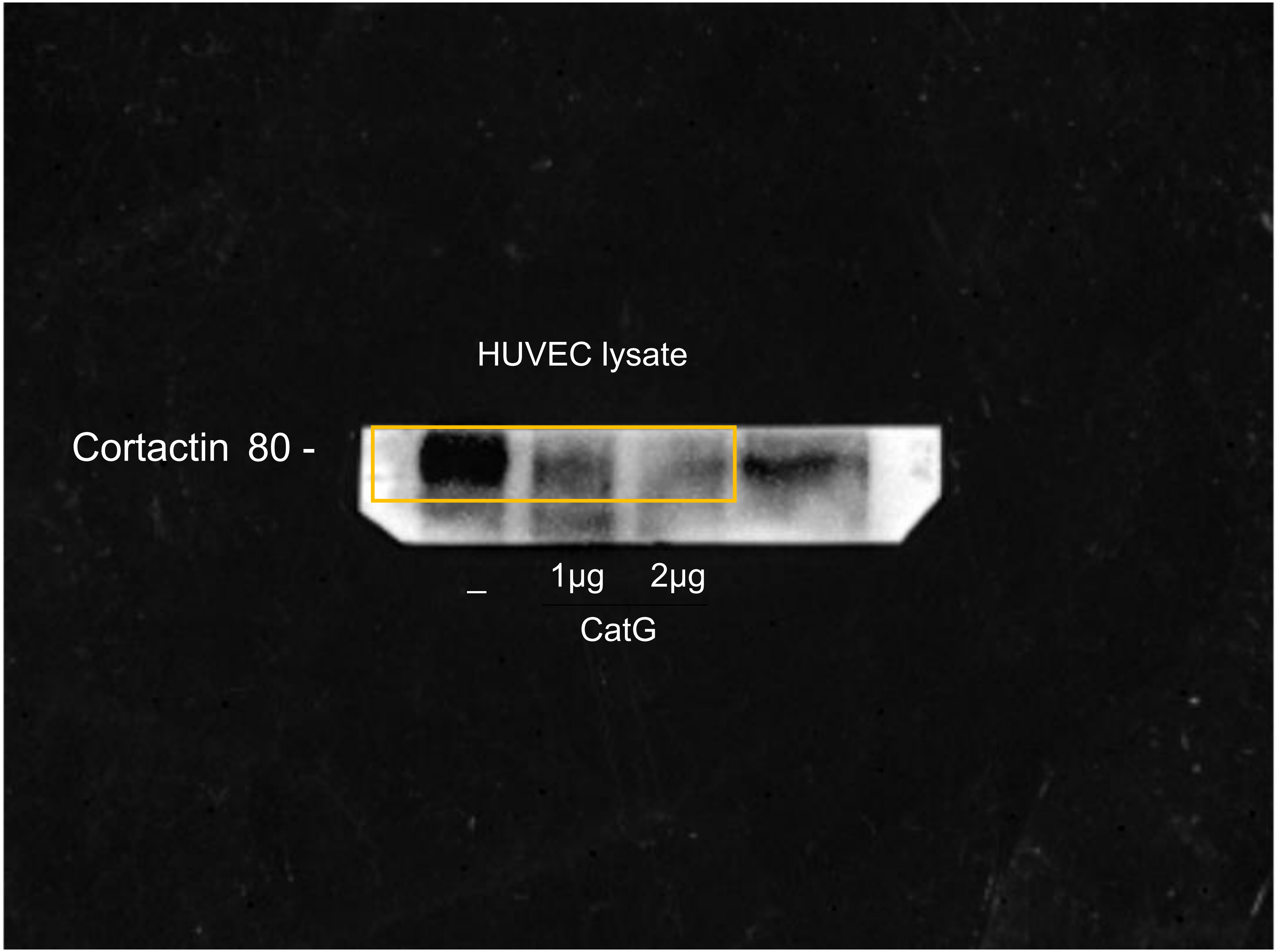
HUVEC lysate
Cortactin 80 -
_
1µg
2µg
CatG

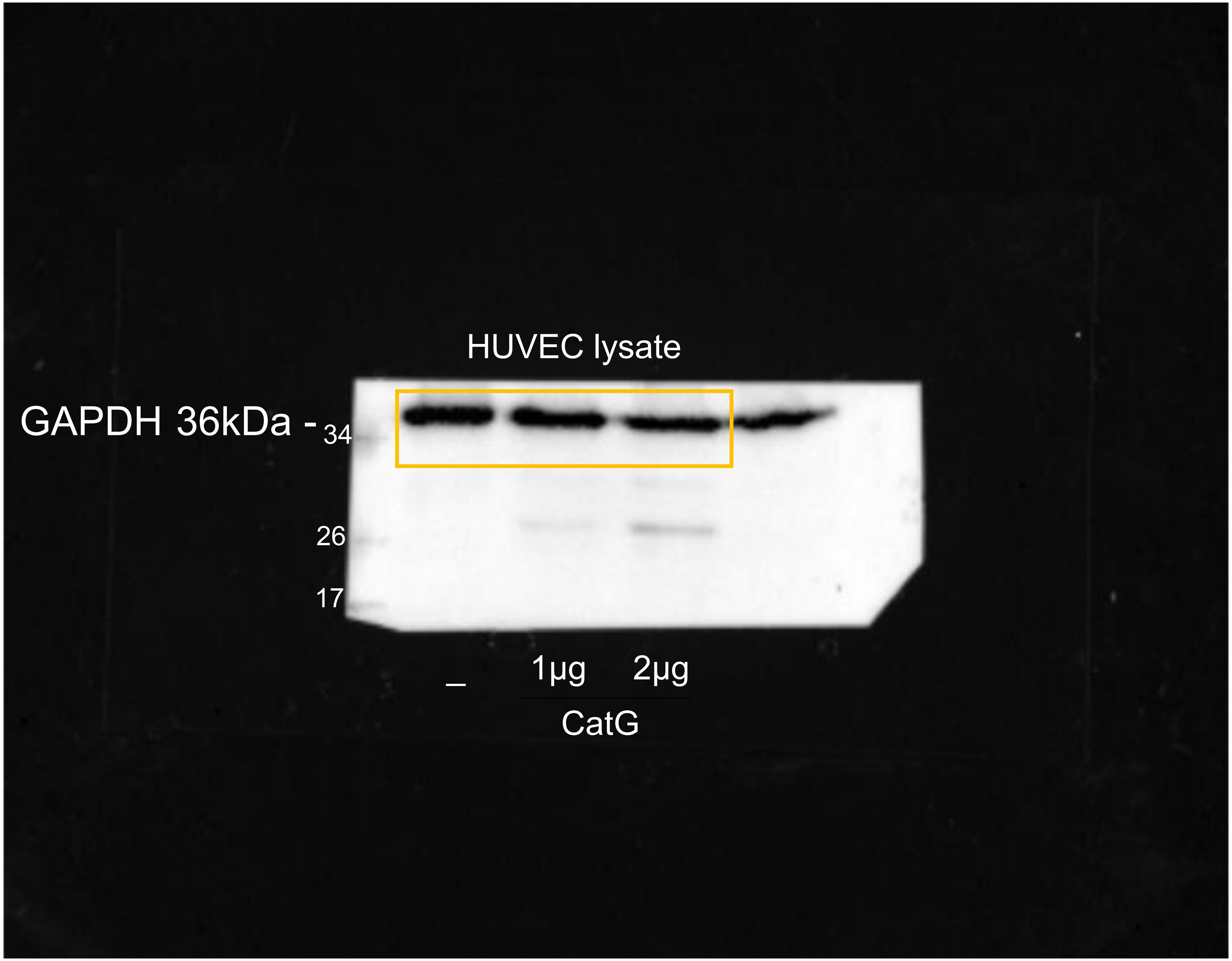
HUVEC lysate
GAPDH 36kDa -
34
26
17
_
1µg
2µg
CatG

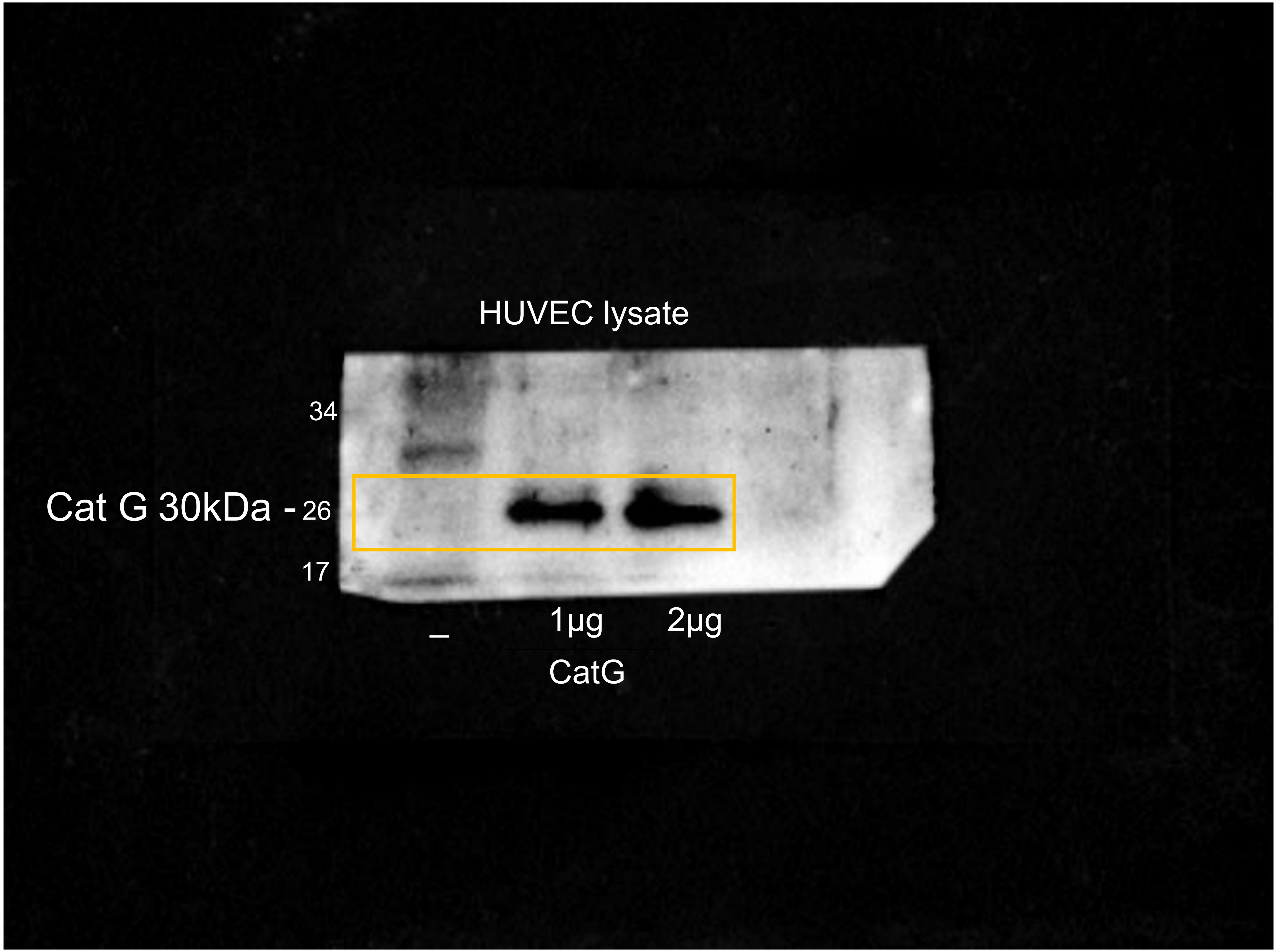

HUVEC lysate
34
Cat G 30kDa - 26
17
_
1μg
2μg
CatG

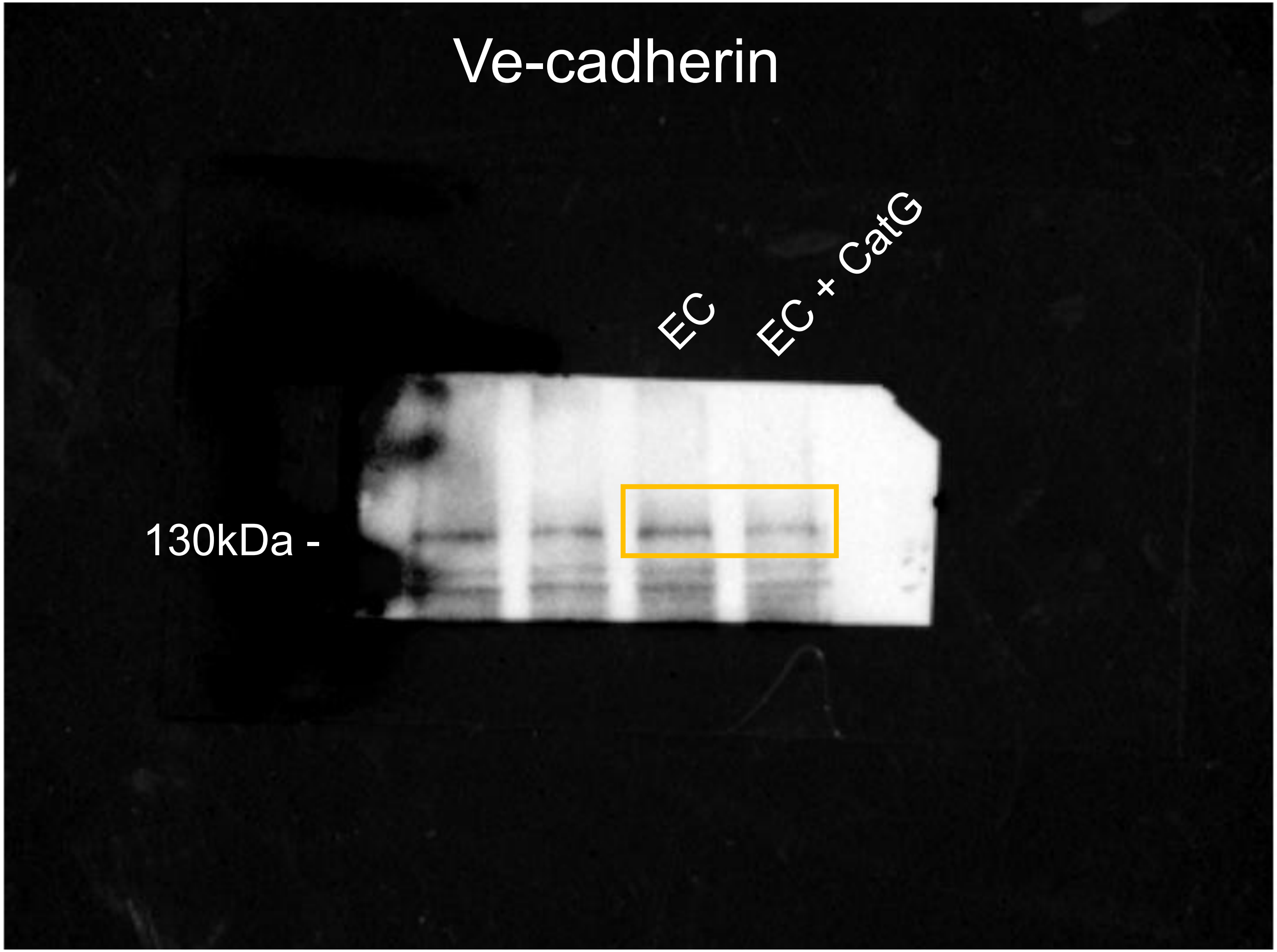
Ve-cadherin
EC
EC + CatG
130kDa -

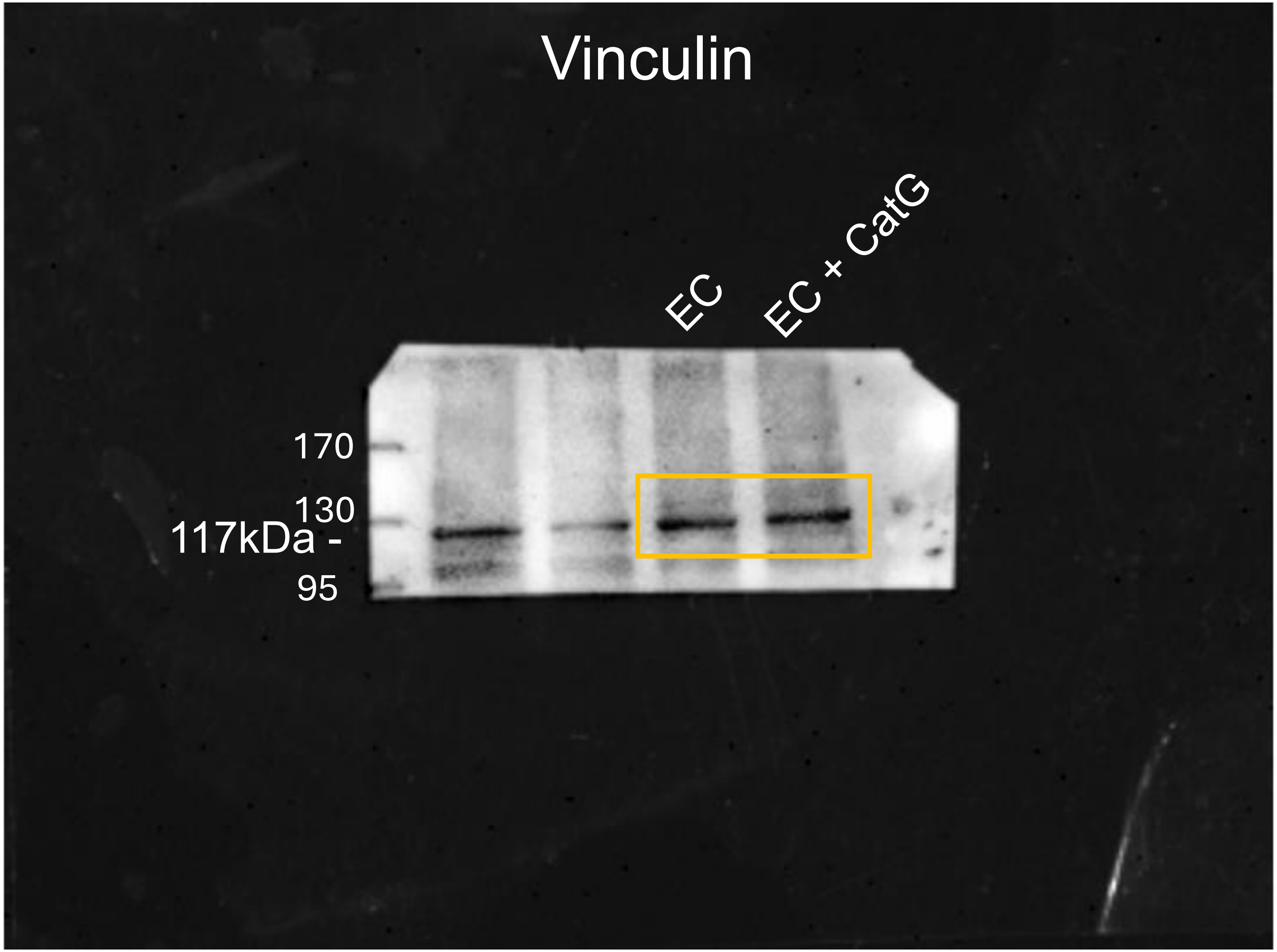
Vinculin
EC
EC + CatG
170
130
117kDa -
95

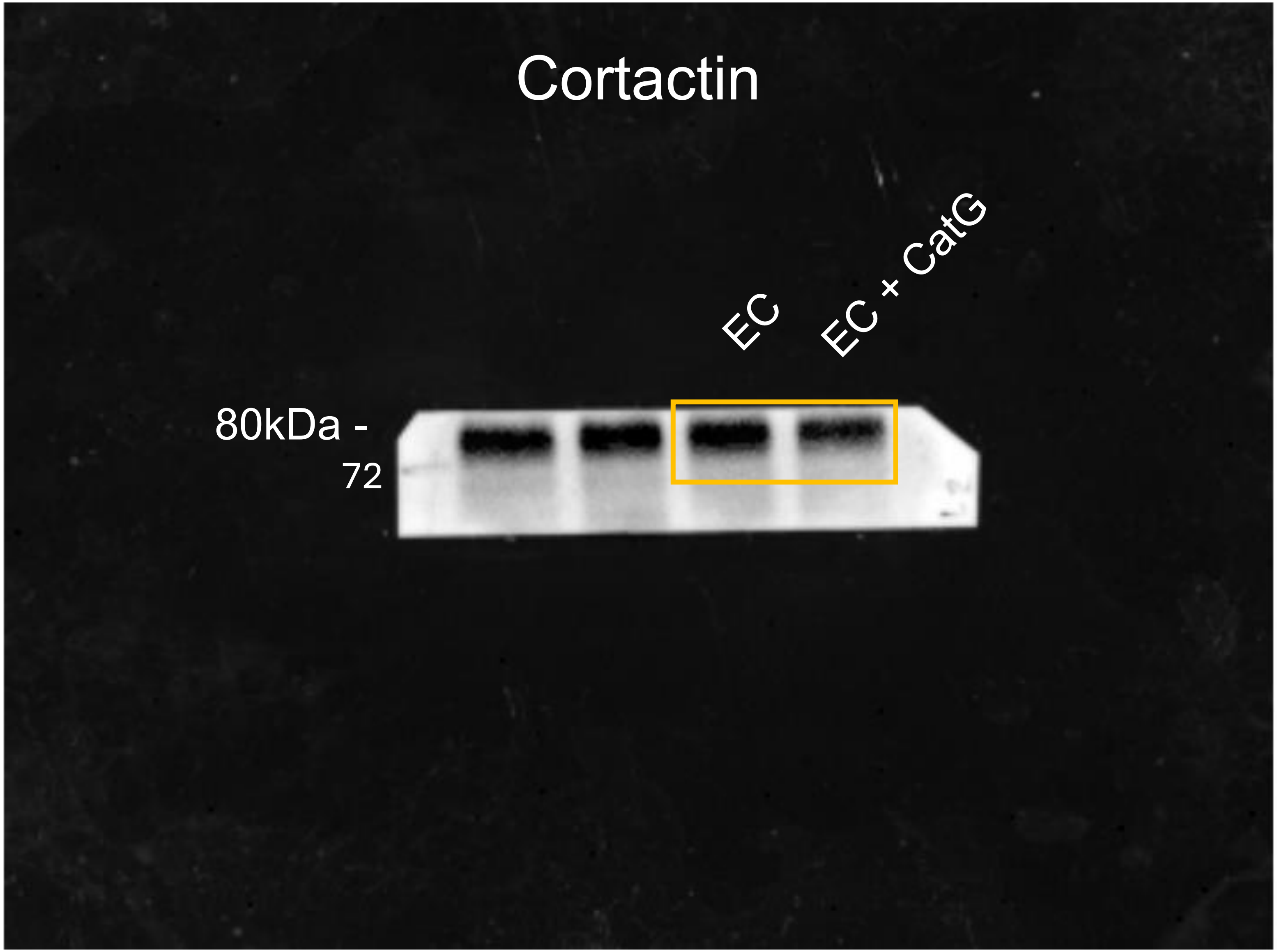
Cortactin
EC
EC + CatG
80kDa -
72

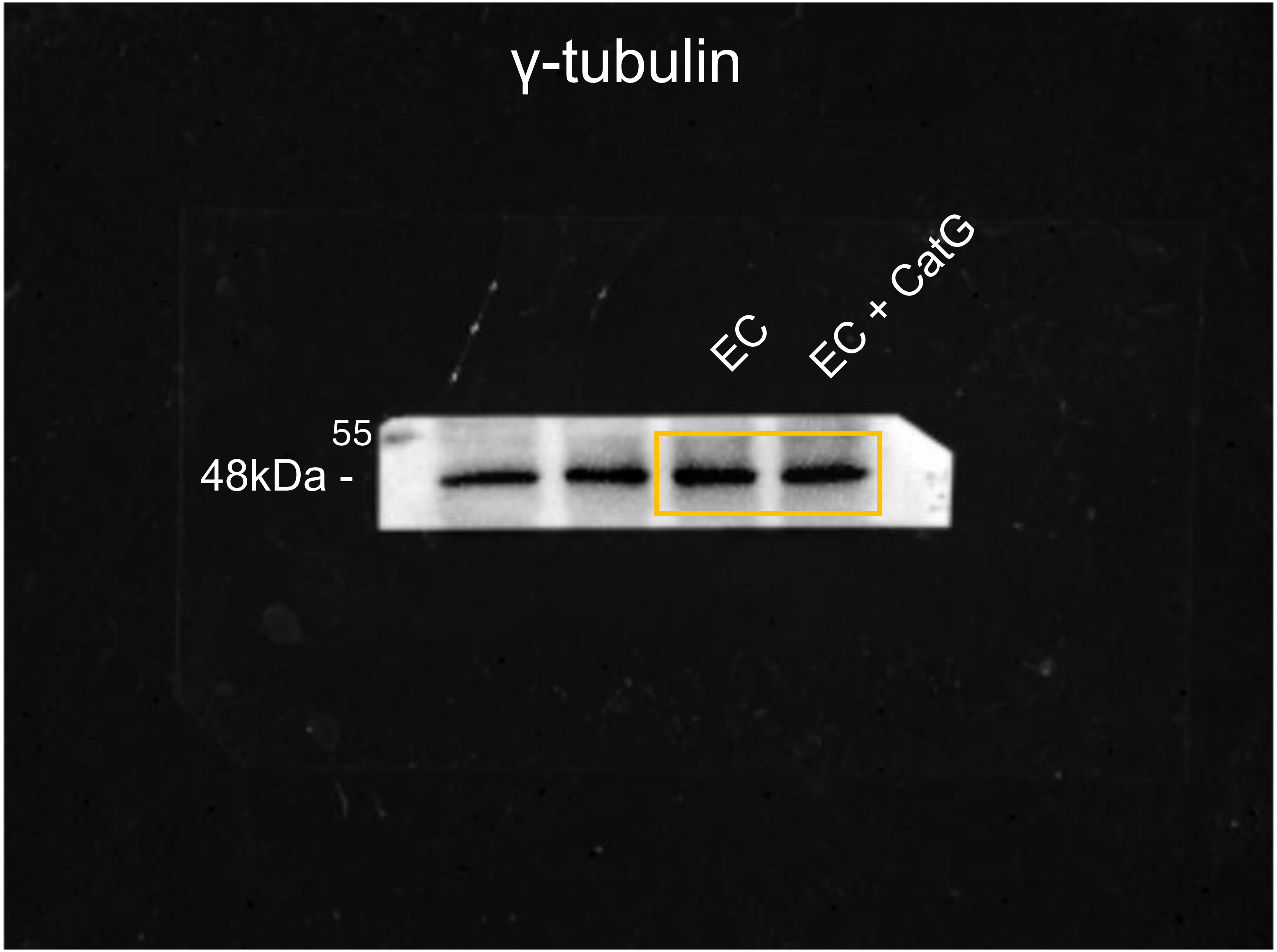
γ-tubulin
EC
EC + CatG
55
48kDa -

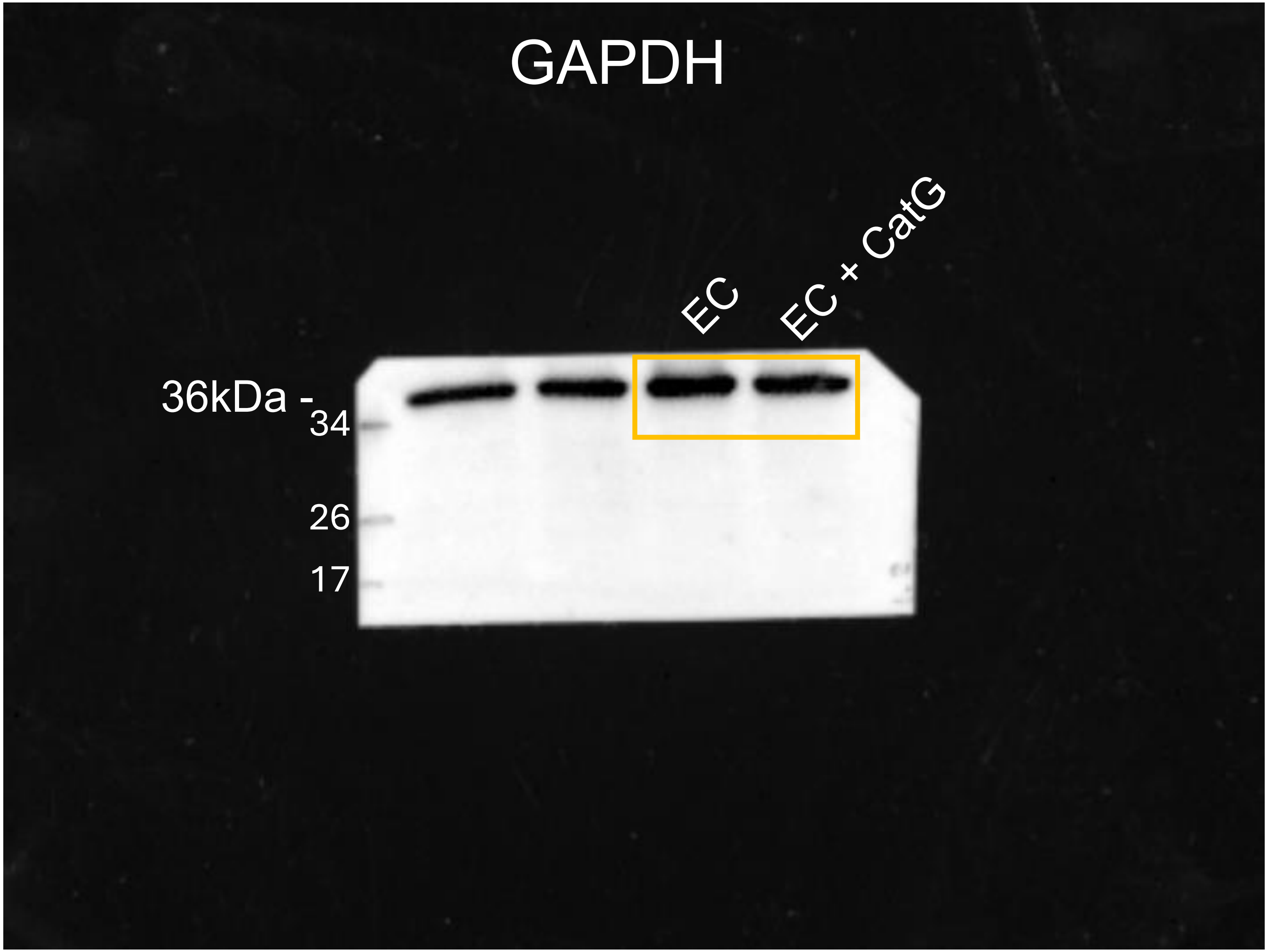
GAPDH
EC
EC + CatG
36kDa -
34
26
17

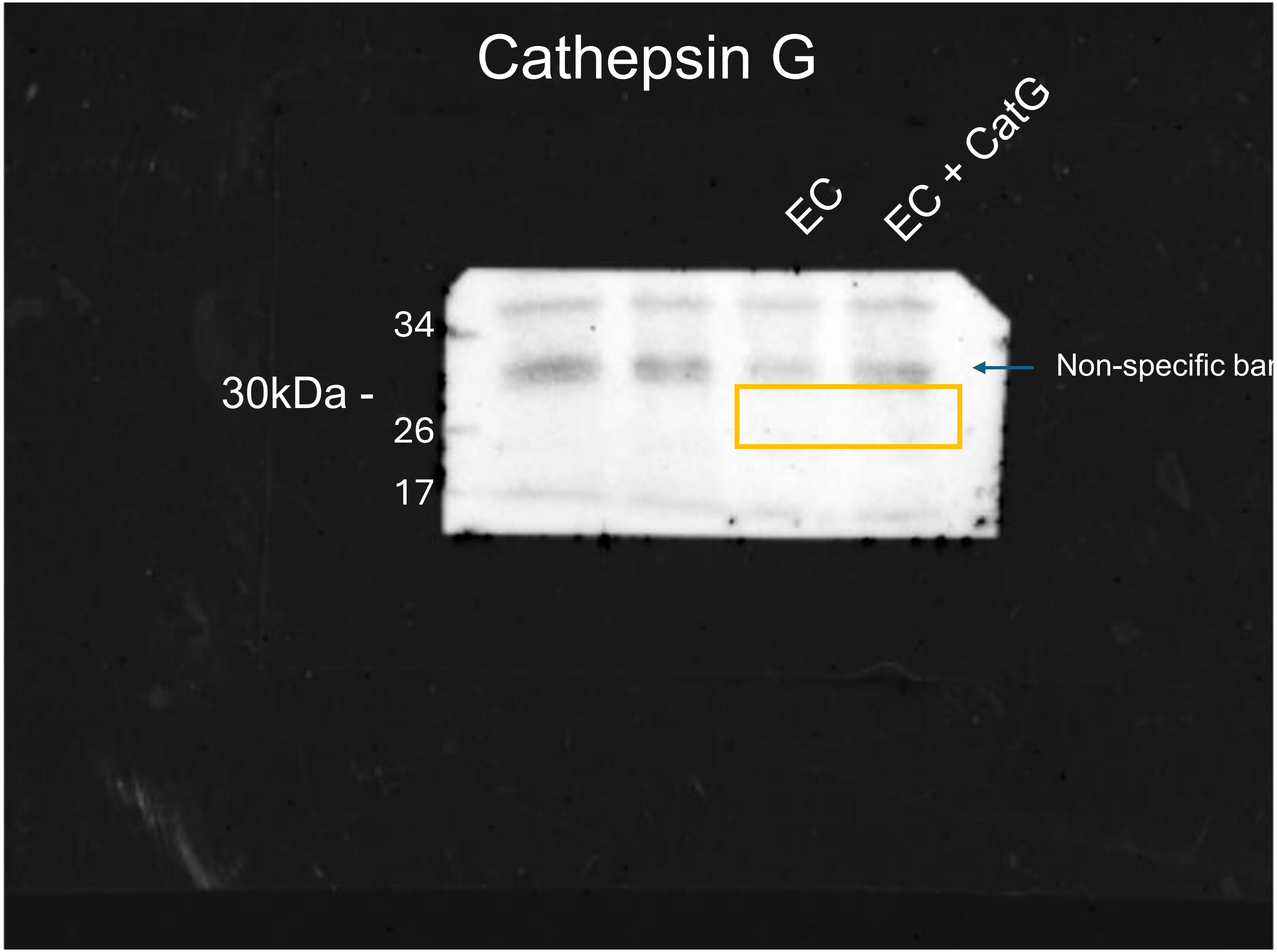
Cathepsin G
EC
EC + CatG
34
30kDa -
26
17
Non-specific bar

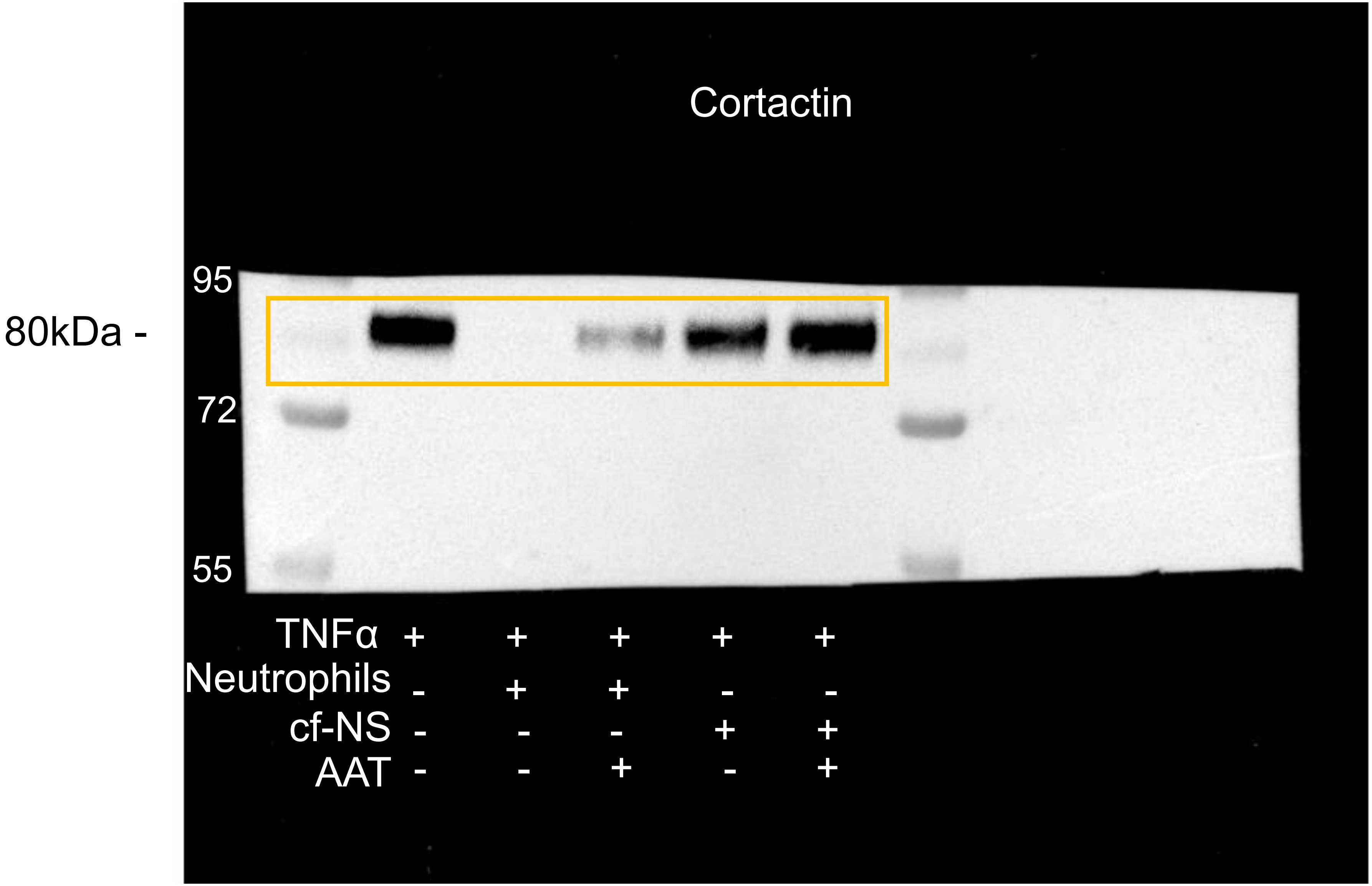
Cortactin
80kDa -
95
72
55
TNFα + + + + +
Neutrophils - + + - -
cf-NS - - - + +
AAT - - + - +

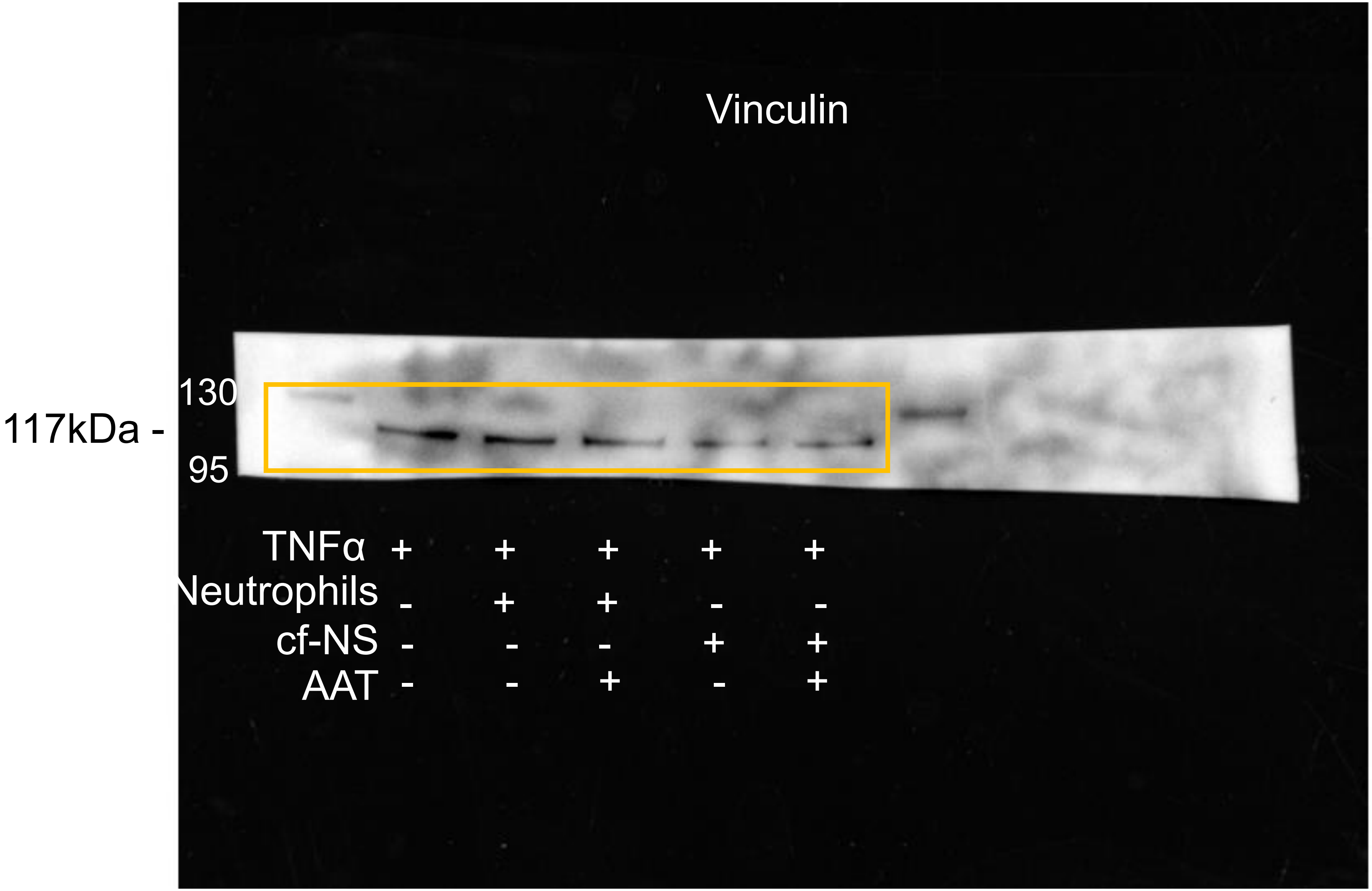
Vinculin
130
117kDa -
95
TNFα + + + + +
Neutrophils - + + - -
cf-NS - - - + +
AAT - - + - +

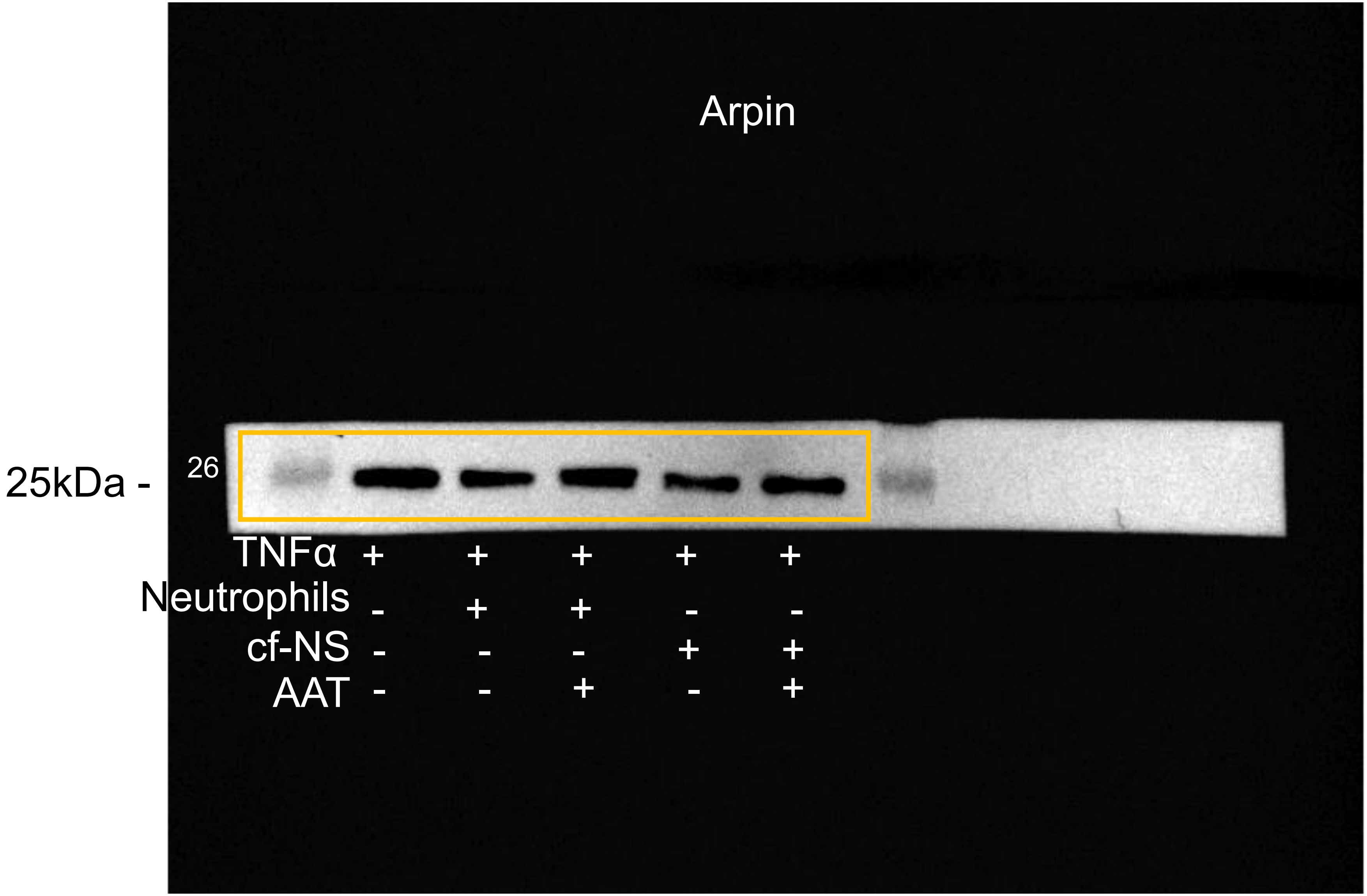
Arpin
25kDa -
26
TNFα + + + + +
Neutrophils - + + - -
cf-NS - - - + +
AAT - - + - +

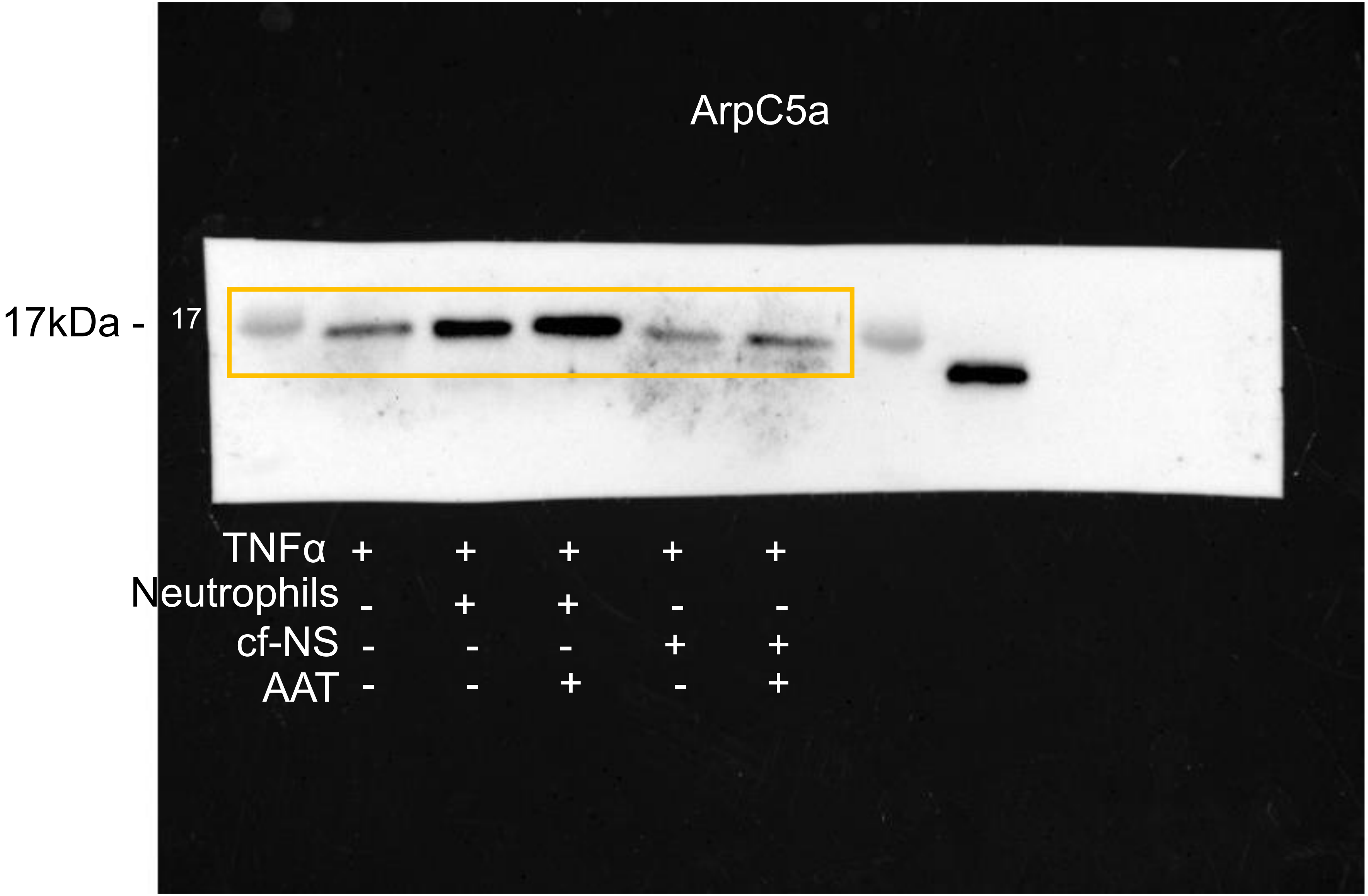
ArpC5a
17kDa -
17
TNFα + + + + +
Neutrophils - + + - -
cf-NS - - - + +
AAT - - + - +

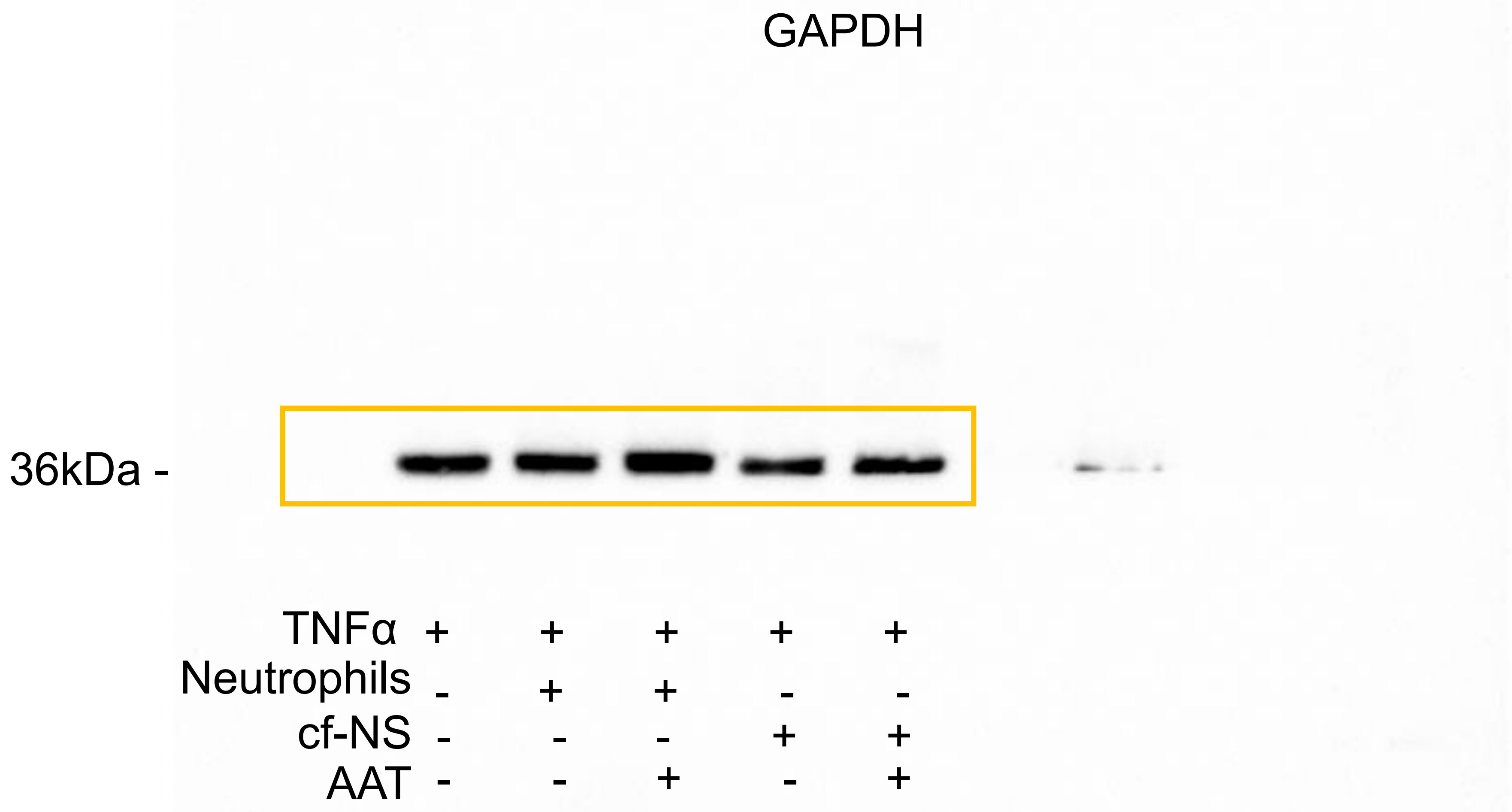
GAPDH
36kDa -
TNFα + + + + +
Neutrophils - + + - -
cf-NS - - - + +
AAT - - + - +

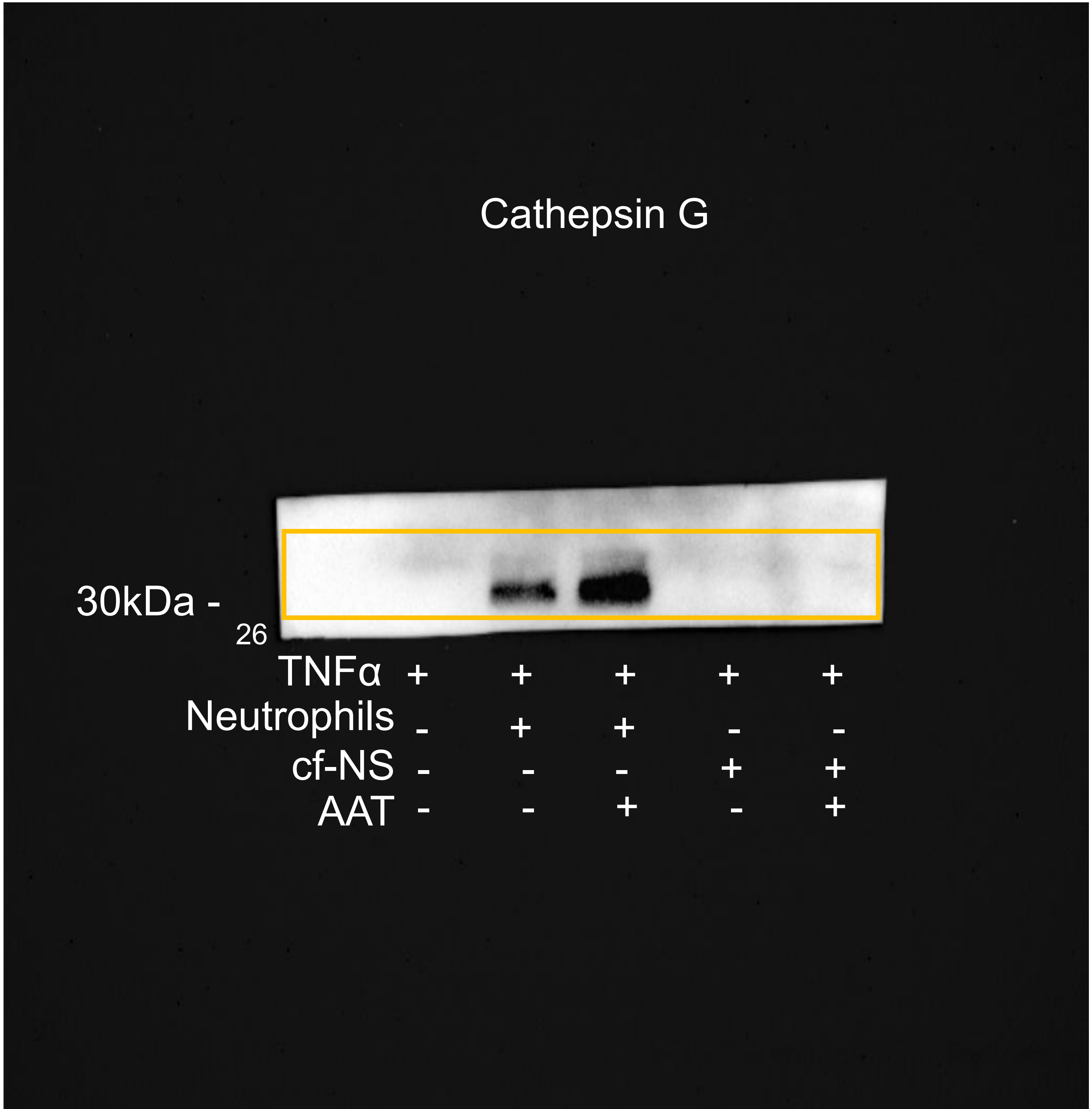
Cathepsin G
30kDa -
26
TNFα + + + + +
Neutrophils - + + - -
cf-NS - - - + +
AAT - - + - +

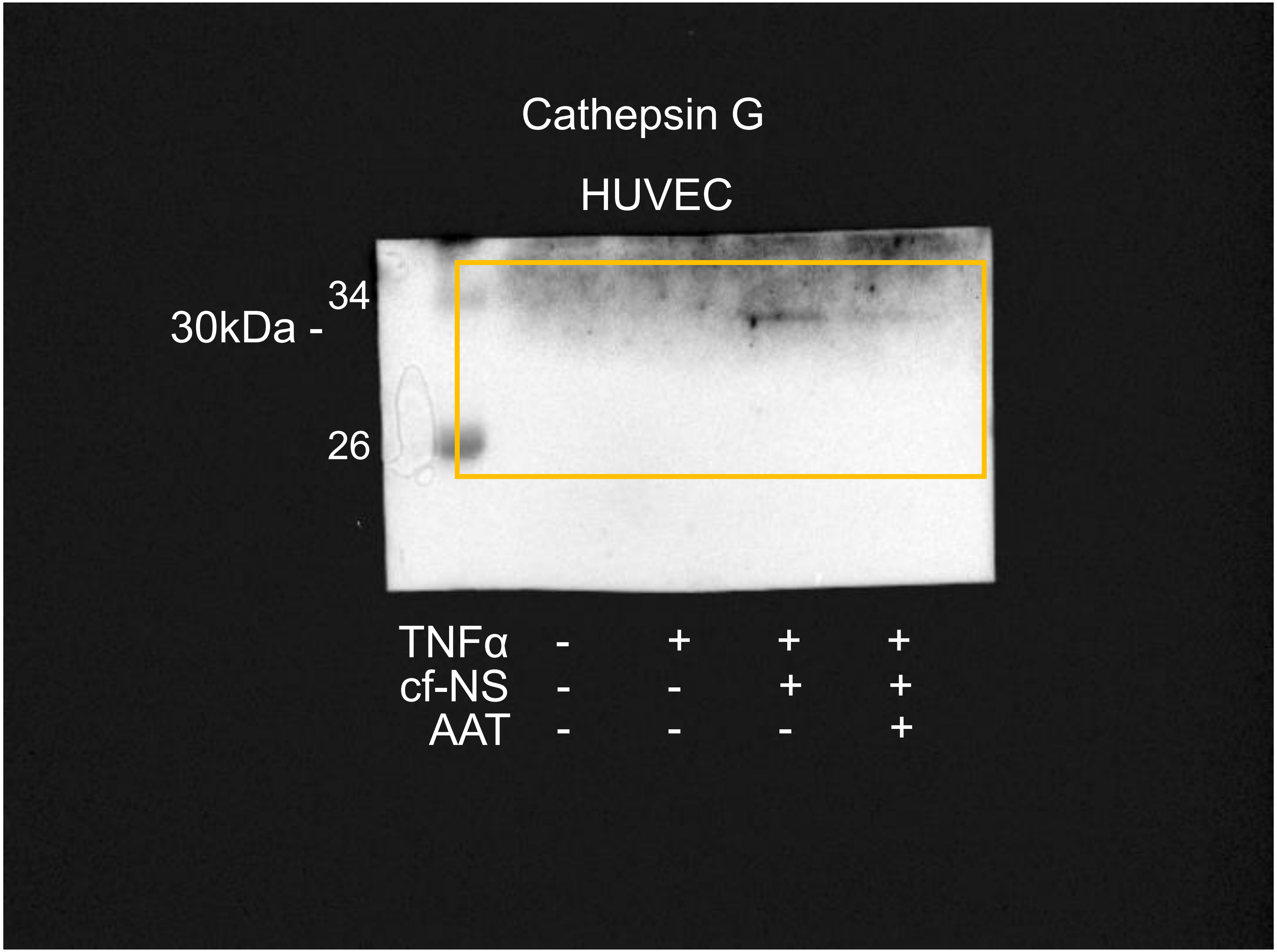
Cathepsin G
HUVEC
34
30kDa -
26
TNFα - + + +
cf-NS - - + +
AAT - - - +

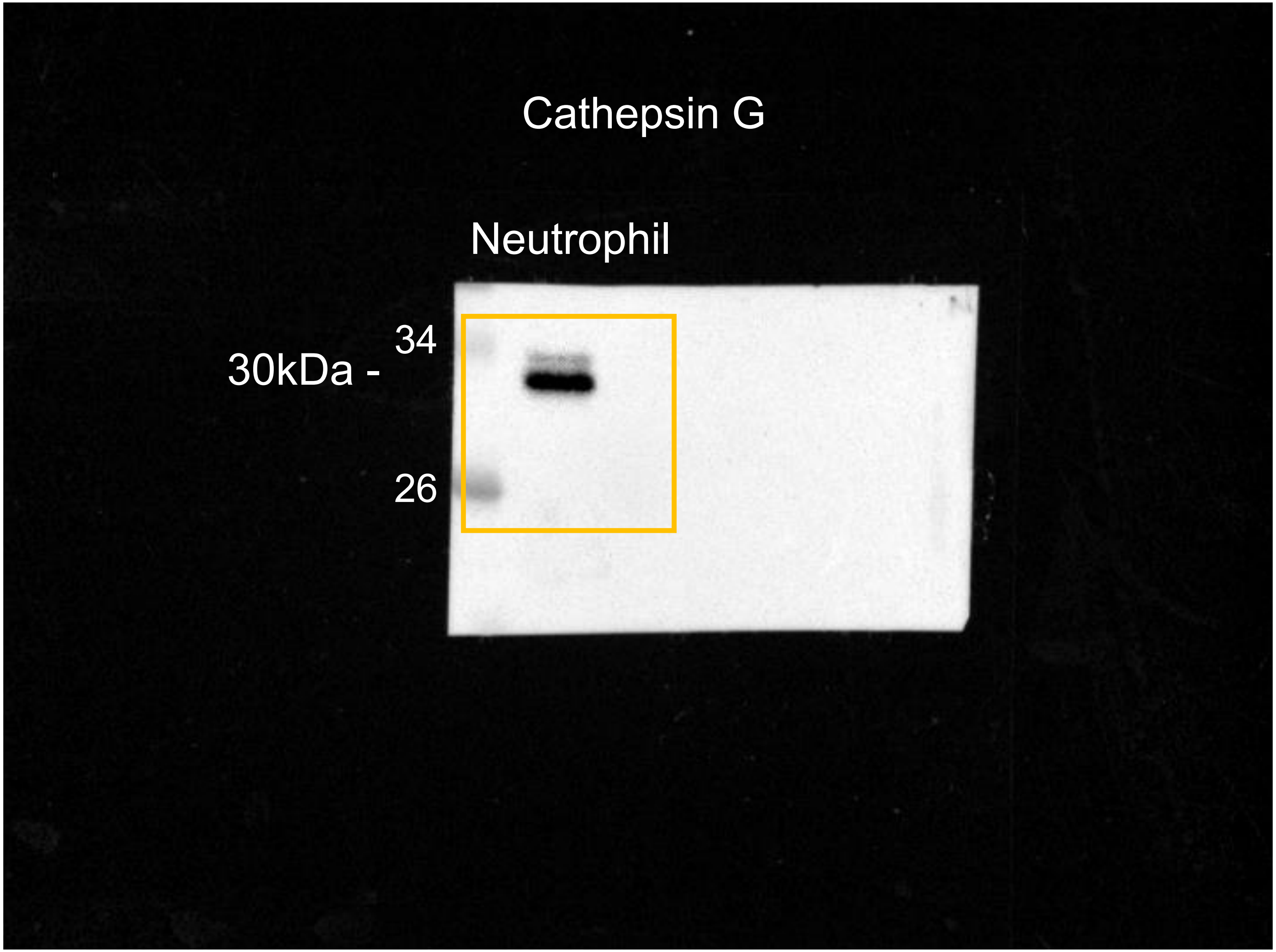
Cathepsin G
Neutrophil
30kDa -
34
26

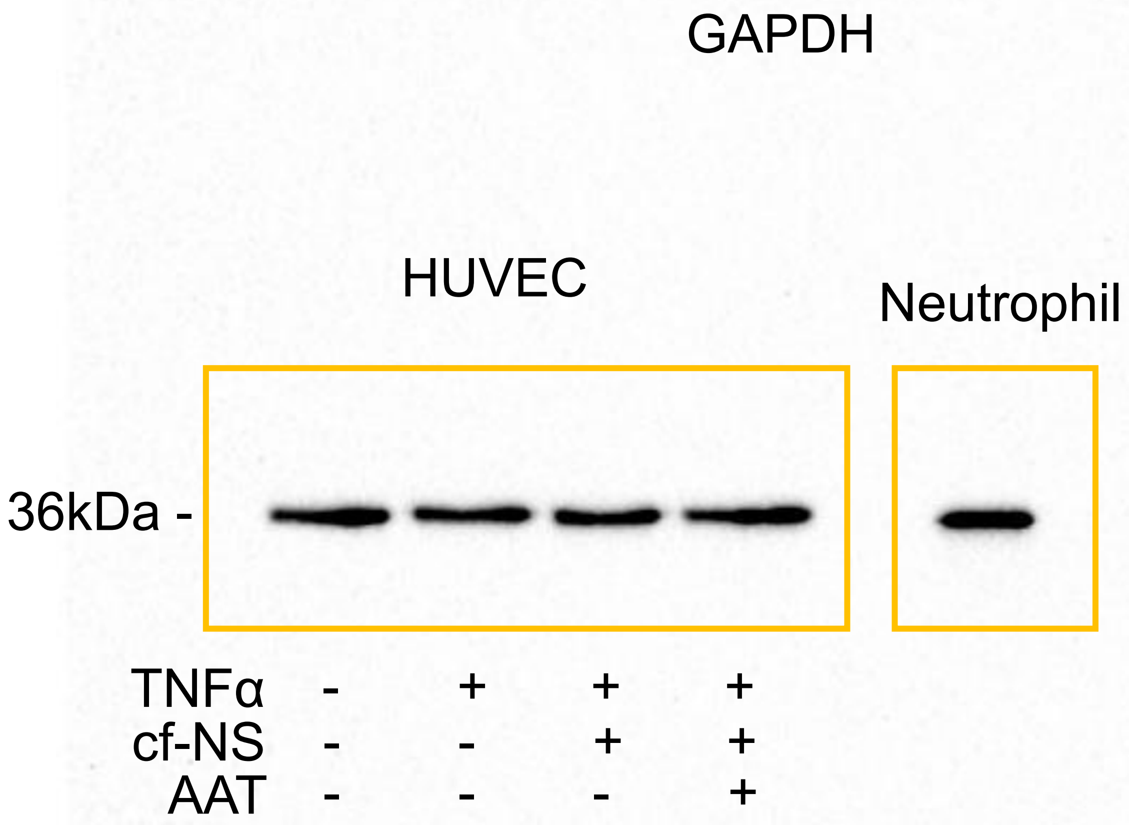
GAPDH
HUVEC
Neutrophil
36kDa -
TNFα - + + +
cf-NS - - + +
AAT - - - +
