# Supplementary figures and images for: Neutrophil serine proteases degrade endothelial cortactin and promote extravasation
Source: J Cell Biol. 2026 May 12;225(7):e202410019. doi: 10.1083/jcb.202410019 (PMC13163681; doi:10.1083/jcb.202410019)

# ICAM-1

HUVEC

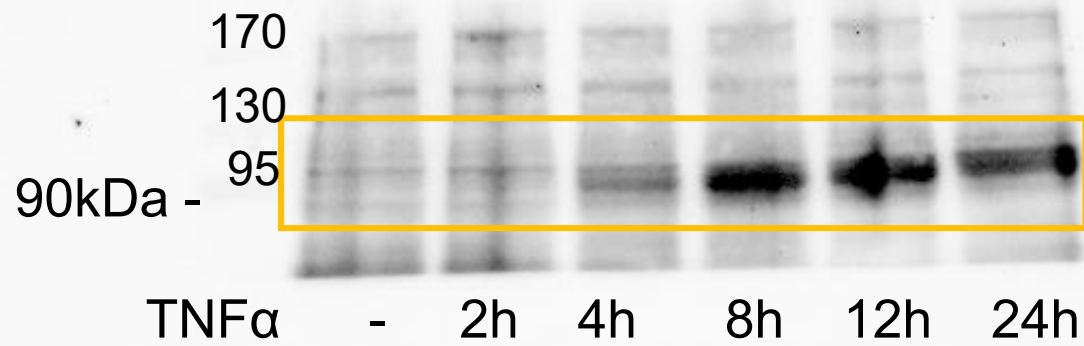

# Cortactin

HUVEC

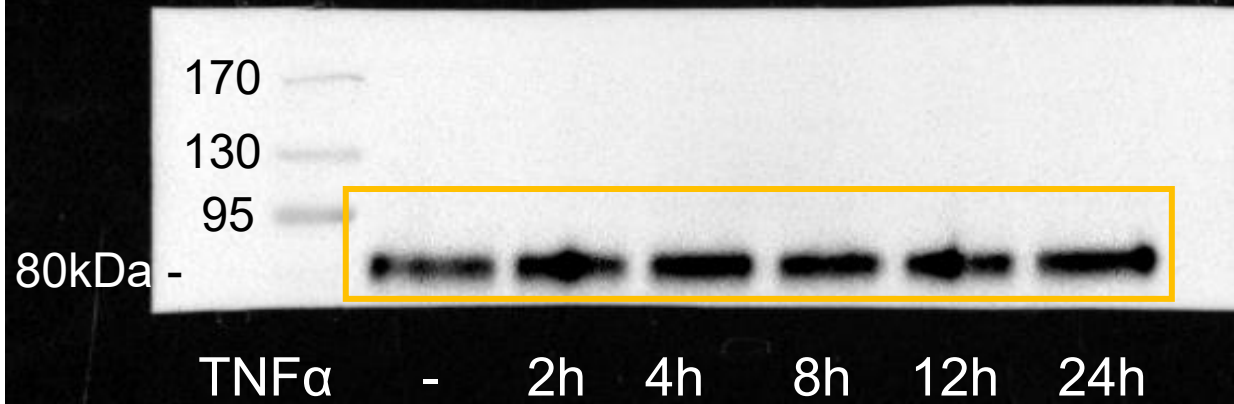

# GAPDH

## HUVEC

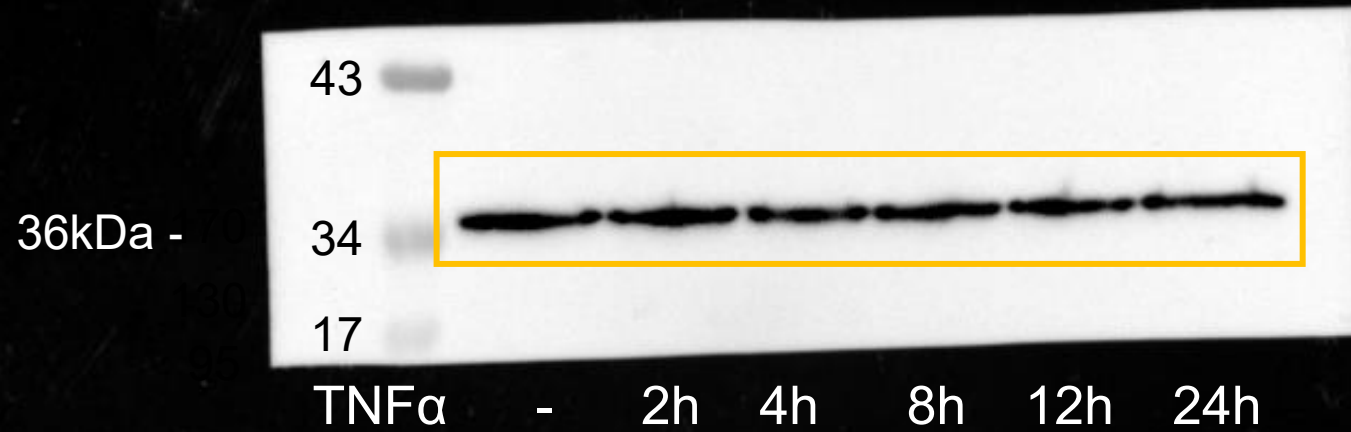

Supplement: SourceData FS2 — is the source file for Fig. S2. [file jcb_202410019_sourcedatafs2.pdf]
